# A Backpack-Mounted Omnidirectional Camera with Off-the-Shelf Navigation Sensors for Mobile Terrestrial Mapping: Development and Forest Application

**DOI:** 10.3390/s18030827

**Published:** 2018-03-09

**Authors:** Mariana Batista Campos, Antonio Maria Garcia Tommaselli, Eija Honkavaara, Fabricio dos Santos Prol, Harri Kaartinen, Aimad El Issaoui, Teemu Hakala

**Affiliations:** 1 São Paulo State University (UNESP), Presidente Prudente 19060-900, São Paulo, Brazil; tomaseli@fct.unesp.br (A.M.G.T.); fabricioprol@hotmail.com (F.d.S.P.); 2 Remote Sensing and Photogrammetry, Finnish Geospatial Research Institute FGI, Geodeetinrinne 2, FI-02430 Masala, Finland; eija.honkavaara@nls.fi (E.H.); harri.kaartinen@nls.fi (H.K.); aimad.elissaoui@nls.fi (A.E.I.); teemu.hakala@nls.fi (T.H.); 3 Department of Geography and Geology, University of Turku, Turku, 20014 Turun yliopisto, Finland

**Keywords:** personal mobile terrestrial system, omnidirectional cameras, low-cost sensors, forest mapping, PMTS data quality

## Abstract

The use of Personal Mobile Terrestrial System (PMTS) has increased considerably for mobile mapping applications because these systems offer dynamic data acquisition with ground perspective in places where the use of wheeled platforms is unfeasible, such as forests and indoor buildings. PMTS has become more popular with emerging technologies, such as miniaturized navigation sensors and off-the-shelf omnidirectional cameras, which enable low-cost mobile mapping approaches. However, most of these sensors have not been developed for high-accuracy metric purposes and therefore require rigorous methods of data acquisition and data processing to obtain satisfactory results for some mapping applications. To contribute to the development of light, low-cost PMTS and potential applications of these off-the-shelf sensors for forest mapping, this paper presents a low-cost PMTS approach comprising an omnidirectional camera with off-the-shelf navigation systems and its evaluation in a forest environment. Experimental assessments showed that the integrated sensor orientation approach using navigation data as the initial information can increase the trajectory accuracy, especially in covered areas. The point cloud generated with the PMTS data had accuracy consistent with the Ground Sample Distance (GSD) range of omnidirectional images (3.5–7 cm). These results are consistent with those obtained for other PMTS approaches.

## 1. Introduction

A Personal Mobile Terrestrial System (PMTS) consists of a set of sensors that are carried by a human operator, for instance in a backpack, to acquire measurements of the environment while walking. The backpack platform provides mobility in environments where wheeled vehicles cannot be used, such as indoor buildings and narrow forest paths. Studies involving PMTS for these applications have increased in recent years.

Indoor mapping aimed at developing a Building Information Model (BIM) was one of the main motivations for the development of embedded systems in a backpack. In 2013, Corso and Zakhor [1] used an arrangement of five LASER (Light Amplification by Stimulated Emission of Radiation) scanners in a backpack platform combined with two fisheye cameras and an Inertial Measurement Unit (IMU) for indoor mapping purposes. The authors achieved centimetric accuracy in three-dimensional (3D) reconstruction. Another backpack platform using a mobile LASER scanning system for indoor and outdoor environments was evaluated in 2015 by Lauterbach et al. [2]. These authors developed a GNSS (Global Navigation Satellite Systems)-free approach based on LASER scanners (SICK LMS 100, and Riegl VZ-400) and a low-cost IMU (Phidgets 1044), with the aim of a simultaneous location and mapping solution (SLAM). A GNSS-free approach using an Extended Kalman Filter (EKF)-based method for indoor environments was also discussed by Wen et al. [3] in 2016. The system proposed by them [3] has three 2-D LASER scanners (UTM-30LX) and an IMU (Xsens MTi-10) specially arranged in a backpack. Commercial solutions with a LASER scanner and omnidirectional systems were recently presented by Leica [4] and Google [5]. The Leica Pegasus Backpack is equipped with a Dual Velodyne VLP-16 LASER scanner and five cameras for a 360° field of view, whereas the Google Street View backpack (Trekker) is composed of an omnidirectional system with 15 cameras, also enabling full 360° imaging.

Another potential application of PMTS is forest mapping. Forest preservation and management requires information about trees and underlying vegetation, such as location, height, volume, stem straightness and Diameter at Breast Height (DBH), to ensure proper management of the forest system [6]. This type of information is used to understand how forests grow and develop, which requires detailed close-range measurements. In 2012, Kukko et al. [7] introduced a backpack personal system composed of a Mobile LASER Scanning system (MLS—FARO Photon 120) and a NovAtel SPAN, which integrates a GPS (Global Positioning System) receiver and an IMU, for forest monitoring in Finland. The authors obtained a 3D point cloud with accuracy in the range of 2–5 cm. However, this PMTS had some disadvantages, such as the need for two operators and the high system weight (approximately 30 kg), which limited its operability. These limitations were reduced by Liang et al. [8] in 2014, with the development of a 10-kg PMTS with a GNSS receiver (NovAtel Flexpak6), an IMU (NovAtel UIMU-LCI) and a FARO Focus3D 120 LASER scanner. This system was applied for forest purposes and estimated DBH with centimetric accuracy. In 2016, a backpack approach with multi digital cameras was presented by Forsman et al. [9]. In this work, a multi-camera system in a backpack structure was developed to estimate tree attributes in low-density forest plots based on terrestrial photogrammetric techniques. This system comprised five synchronized cameras: three Canon EOS 7D with Sigma fixed lenses and two Canon EOS 40D with Canon zoom lenses. The cameras were specially positioned to improve redundancy and to reduce the risk of false matching and occlusions. The process of indirectly estimating DBH with this system contains some sequential steps: cameras and system calibration, image acquisition, image processing, matching using SIFT and RANSAC methods, and circle fitting to estimate tree diameter. DBH was obtained with a Root Mean Square Error (RMSE) of 3.9 cm. Another portable system with multi digital cameras was presented by Tseng et al. [10], also in 2016, in which eight single-lens cameras (Sony NEX-5N) and a GPS receiver were used. More accurate results for forest mapping were presented recently (2017) by Oveland et al. [11]. In this work, a Velodyne VLP16 LASER scanner and an Applanix APX-15 UAV system were assembled in a backpack to capture GNSS and IMU data for forest data acquisition. After data processing, DBH was estimated with an RMSE of 1.5 cm.

The works mentioned above show the relevance and increasing number of studies involving PMTS in backpack platforms in recent years. Some research trends can be highlighted: (1) the use of off-the-shelf navigation sensors [2,3,11], which can be combined in PMTS with (2) optical sensors [4,5,9,10] and (3) the employment of mobile LASER scanner solutions in the PMTS [1,2,3,4,5,6,7,8,11], particularly motivated by recent MLSs, such as the Velodyne Puck LITE and Riegl miniVUX-1UAV. Considering these trends, this paper aims to contribute to the following issues. 

First, despite the diversity of off-the-shelf navigation sensors that have entered the market and the increasing use of these sensors in Mobile Terrestrial Mapping Systems (MTMS) and personal navigation, uncertainties about the data accuracy of these sensors remain. In this regard, this paper presents an overview, experiments and discussions of the data accuracy of the low-cost system used and highlights its potential and limitations. Second, although MLSs provide fast data acquisition, dense surface coverage, high relative accuracy and real-time point cloud visualization, some limitations remain. MLS is still a high-cost system and produces a huge amount of data, which could be a further problem for data processing and long-term storage. Furthermore, MLSs require an accurate navigation system, and real-time data production and visualization requires accurate Real Time Kinematic (RTK) positioning with base stations nearby. The use of omnidirectional systems [12], especially with multi cameras, has also garnered attention due to significant improvements in recent years, including lighter, smaller, lower cost and more diverse commercial models (Giroptic 360cam, Go Pro 360°, Kodak PixPro SP360, Lady bug5, Google Jump system, Ricoh Theta S, Samsung Gear 360, LG 360 and Nikon KeyMission 360). Omnidirectional sensor solutions can outperform other sensors, such as LASER scanners, in terms of field of view, the amount of usable information from the environment for mapping, simple operating principles and intuitive data interpretation [13]. However, research focused on PMTS using optical systems, especially those based on omnidirectional cameras, are still underexplored [9,10]. In this regard, this paper presents the description and assessment of a PMTS with a backpacked omnidirectional system and off-the-shelf navigation sensors for forest mapping. 

## 2. An Omnidirectional Backpack Mobile Image System 

### 2.1. PMTS Setup

**Figure 1:**
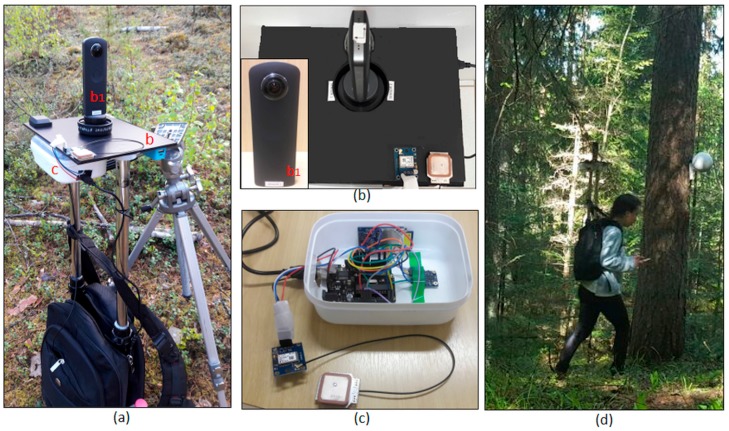
Personal Mobile Terrestrial System (PMTS): (**a**) backpack platform structure and PMTS sensors; (**b**) top view of platform with the omnidirectional camera and GPS receiver; (**c**) navigation sensors; and (**d**) example of an operator carrying the PMTS inside a forest.

Figure 1 presents the proposed PMTS. The system components cost approximately $450 and weighed 3 kg. The backpack was equipped with a rigid structure in a “T” design, thereby enabling sensor assembly in the upper and bottom parts of the structure (Figure 1a). The platform was retractable and could be stored inside the backpack for practical transportation.

The omnidirectional system was positioned in the top of this structure with the GPS receiver and antenna (Figure 1b), whereas the IMU, Arduino microprocessor and SD (Secured Digital) card (Figure 1c) were placed below to avoid image occlusions. An example of an operator carrying the backpacked system inside a forest area is presented in Figure 1d. The PMTS can be divided into two sets of sensors: an optical imaging sensor (Ricoh Theta S camera) and navigation sensors (IMU and GPS). Section 2.1.1 presents the imaging sensor configuration and the camera calibration methodology applied, whereas Section 2.1.2 shows the navigation sensor settings.

#### 2.1.1. Omnidirectional System

Omnidirectional images can be obtained using panoramic cameras, fisheye lenses, poly-dioptric solutions and catadioptric systems [12]. The omnidirectional system used in the PMTS is a poly-dioptric camera named Ricoh Theta S. A poly-dioptric spherical imaging system can be defined as a combination of multiple cameras in the same structure to cover a full-spherical field of view, acquiring multiple frames simultaneously [12]. These systems have earned attention due to their portability and wide coverage. Ricoh Theta S is a combination of two CMOS (Complementary Metal-Oxide Semiconductor) digital cameras with fisheye lenses that are specially arranged in a back-to-back position to provide a 360° field of view. Table 1 shows the Ricoh Theta S camera settings [14].

The Ricoh Theta S data can provide either still images or dual-fisheye video. The still image mode requires an 8-s processing time between successive image shots. This time delay for image acquisition and processing is not convenient when considering the movement during a PMTS survey and will hinder an appropriate base geometry and later reconstruction by bundle adjustment. Furthermore, uncertainties in the accuracy of image stitching remain that are not easily modeled. Since the stitching parameters are unknown, further errors may accumulate in the photogrammetric processing [15,16].

Considering these limitations when using the still mode of the Ricoh Theta S, the dual-fisheye video mode was used to acquire omnidirectional data to provide dense and continuous frame acquisition. Furthermore, the dual-fisheye video is stored in the camera without any internal processing. The Ricoh Theta S can record 65 min of dual-fisheye video in a 1920 × 1080 resolution mode, which is enough to collect data, for instance, from a standard forest plot with 32 m × 32 m dimension. PMTS operability time is directly associated to the camera internal battery and storage. The main disadvantage of the dual-fisheye video, compared to the still images, is its low resolution. Ricoh Theta S has an Instantaneous Field Of View (IFOV) of 0.2°. Therefore, the 3D reconstruction of objects that are located farther than 5 m from the camera—at the optical axis direction—presents inaccurate results, especially due to the low level of detail of these objects (GSD > 1.7 cm). Improving the camera resolution will also augment this distance.

**Figure 2:**
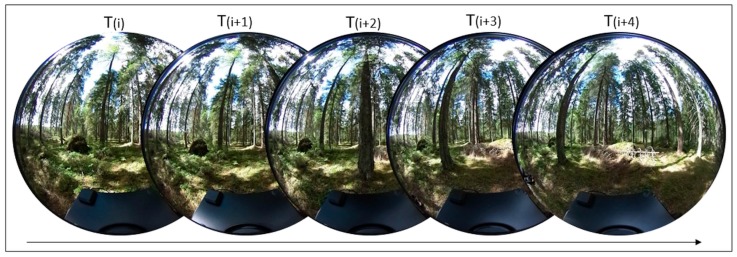
Example of sequential omnidirectional images acquired in a forest area using the Personal Mobile Terrestrial System.

The dual fisheye frames extracted from the Ricoh Theta S video can be converted automatically (with a MATLAB Script) into individual frames for each camera that composes the Ricoh Theta S, with dimensions of 960 × 1080 pixels and a pixel size of 0.005 mm. These individual frames will be called omnidirectional images in this paper. Figure 2 shows an example of an omnidirectional image sequence used as input data in the subsequent photogrammetric processes.

A system calibration is needed to model the internal geometry of the Ricoh Theta S imaging system. The calibration requires the estimation of two sets of Interior Orientation Parameters (IOP) and Relative Orientation Parameters (ROP). Fisheye lenses usually have a short focal length, 1.43 mm in the Ricoh Theta S, and a very wide field of view (~180° of coverage). Therefore, the geometry of a fisheye lens can be modeled by projecting the three-dimensional object onto the surface of a sphere and then reprojecting this sphere to the image plane [17,18]. The projection of the points on the sphere surface into an image plane can be achieved by applying fisheye projections, such as stereographic, equidistant, orthogonal and equi-solid-angle projections [18]. The Ricoh Theta S fisheye lenses follows an equidistant projection [14]. An equidistant projection function with a Conrady–Brown distortion model [19] was chosen as the mathematical model for the camera calibration process, after an experimental assessment. For IOP and ROP estimation, a simultaneous bundle adjustment with relative orientation stability constraints was performed [16] using the unified approach to least squares adjustment [20]. More details about this camera calibration methodology and results can be found in Campos et al. [16]. 

#### 2.1.2. Navigation System

The navigation system is composed of a GPS receiver (Ublox NEO-6M) and an IMU (MPU 6050) integrated with an Arduino microprocessor (Blackboard, produced by RoboCore), and the sensor data are recorded with an SD card adapter. The navigation sensors’ properties are summarized in Table 2. 

Ublox NEO-6M is a low-cost single frequency GPS receiver with a compact architecture and low power consumption, which is ideal for battery-operated systems. Like most low-cost GPS receivers, this device has an internal GPS active antenna designed for tracking L-band signals in the frequency of 1575 MHz. The processed data are delivered in the NMEA (National Marine Electronics Association) protocol, which provides the position, velocity and time (PVT) data computed by the receiver and detailed information on the satellites tracked. In addition, the NEO-6M supports Differential GPS (DGPS) operation using RTCM (Radio Technical Commission for Maritime Services) correction messages from a local reference station or aiding network. Alternatively, raw GPS observations can be accessed by sending specific messages from RTKlib [21], which provides the user with many options for obtaining coordinates, with the goal of activating a low-cost mobile mapping solution. Other receivers from Ublox, such as NEO-6T and NEO-6P, provide GPS raw data output and may be better options depending on the user application.

The positions retrieved with the low-cost GNSS receivers are mainly affected by the atmospheric refraction, errors on the broadcast ephemeris, multipaths and measurement noise. Such errors and the use of pseudoranges create a difficult scenario for achieving sub-metric accuracy. However, its affordable cost, small size, and low power consumption have inspired many efforts in the development of techniques and algorithms to improve its positional solution. For instance, Takasu and Yasuda [21] developed a low-cost unit linked to a high-precision algorithm for performing RTK positioning and obtained a centimetric error with a reasonable fixing ratio of 50–60% using a baseline length of 7 km. Hedgecock et al. [22] also developed a differential algorithm for short baselines to reach a relative positional error of approximately 10 to 35 cm, depending on the speed of the receiver. These studies were performed in areas with unobstructed sky conditions. For forests, challenging conditions, such as distortion and attenuation or obstruction of the GPS signals by branches, trunks and leaves, must be considered. In this regard, some studies have been performed to estimate low-cost GPS accuracy in forest environments [23]. These studies have provided meter-level positional accuracy driven by forest canopy characteristics, such as the stand density. These good results inspired an evaluation of Ublox NEO-6M positional accuracy in both open-sky and forest environments, which is presented in Section 4.1.

The inertial measurement unit used, named MPU 6050, is a combination of three axis MEMS accelerometers and MEMS gyroscopes (six degrees of freedom—6DoF) integrated in a GY-521 electronic board. MPU 6050 provides accelerations and gravity data for the *X*, *Y* and *Z* axes using a 16-bit digital analogue converter. Then, the angles (yaw, pitch and roll) are estimated in real time using an EKF approach. With the aim of acquiring more suitable data from MPU6050, a previous calibration to estimate the three gyroscopes and three accelerometer offsets was performed using a I2Cdev device library code [24]. The six parameters estimated in the IMU calibration were used in the main Arduino script for data integration (Section 2.2).

Some works have focused on the feasibility analysis of MPU 6050 applications in mobile mapping. Alam et al. [25] presented a comparison of MPU6050 performance using complementary filters, for instance, the explicit complementary filter (ECF) and gradient descent-based orientation filter (GDOF), and showed improvements in terms of time processing and angle estimation. Marino et al. [26] presented an application using the MPU 6050 in a vehicle platform. These authors assessed the accuracy of the acceleration measurement with this IMU over time of trajectory and obtained a standard deviation of 0.1921 m/s^2^. The performance of a low-cost MEMS-based orientation sensor associated with a Kalman filter and a digital imaging sensor has been also analyzed for the estimation of a two-wheel vehicle trajectory, showing meter-level accuracy in position estimation [27]. The behavior of motorcycles resembles that of a PMTS system since in both cases a significant variation in roll angle can be observed that is not observed for four-wheel vehicles. These variations affect the estimation of the yaw and pitch angles [27]. In addition to these studies, the accuracy of the trajectory obtained with MPU 6050 in the PMTS is discussed in Section 4.1. 

### 2.2. PMTS Data Integration

PMTS provides omnidirectional images, GPS positions and orientation angles from the IMU. Time synchronization and estimation of position and angle offsets between each measurement unit are therefore required to extract the corresponding attitude and position of the camera for each instant in which images were acquired. This section describes the procedures from acquisition through post-processing for data integration.

An Arduino script was developed to synchronize the navigation sensors. The algorithm starts by defining the sensor configuration. MPU 6050 and Ublox NEO-6M were set to a baud rate of 9600 bps (bits per second). The GPS receiver had a data collection rate of 1 Hz, and the IMU data collection rate was 10 Hz. After the configuration settings, both sensors are initialized. In the case of signal or connection loss, the sensor initialization function is restarted. The main loop was developed to collect NMEA GPS and IMU raw data, compute the yaw, pitch and roll angles (*y, p, r*) from the IMU raw data using EKF, and record both on the SD card. The Arduino microcontroller communicates with the SD card using a Serial Peripheral Interface (SPI). Navigation data were recorded on the SD card considering a GPS data frequency with a rate of 1 s. 

One difficulty in PMTS data integration is the communication between the omnidirectional system and the navigation system, since Ricoh Theta S does not provide a serial port for this purpose. In this regard, the Coordinated Universal Time (UTC) obtained from Ublox NEO-6M was used for sensor data integration. The Ricoh Theta S records 29 dual-fisheye video frames per second, and the camera clock was set in Local Time (LT). Therefore, frame videos could be associated with the navigation data considering the transformation between LT and UTC. Visual synchronization was implemented by means of a small LED (Light Emitting Diode). The light pulse was synchronized with the GPS record and appeared in the dual-fisheye video frames. This synchronization was performed with an average error of 0.04 s. A typical person walks at an average speed of 1.4 m/s, resulting in, at most, a 5-cm synchronization error in the object space, which is less than the expected positioning error using low-cost GPS receivers [21,22]. 

Another concern is the estimation of linear and angular misalignments between sensors to fully calibrate the system. PMTS calibration consists of two main steps: first, ROP is estimated between cameras in the omnidirectional system, which has been performed in the camera calibration process considering the Exterior Orientation Parameters (EOP) estimated in the bundle adjustment [16]. Then, the parameters between the cameras and navigation system sensors are estimated, which consist of lever-arms (offsets) between the camera projection centers and GPS antenna phase center and boresight misalignments between the IMU and cameras. 

Since ROP between cameras are known, the lever-arm and boresight can be calculated for camera 1 and extended to camera 2. Several methods have been proposed to estimate system calibration parameters with direct and indirect measurement approaches. Regardless of the method used to estimate the lever-arms and boresight angles, the accuracy of these parameters should be superior to the system noise level. Therefore, considering an expected centimetric accuracy for PMTS data, the lever-arms (offsets) between each camera projection center and GPS antenna phase center (Δ*x*, Δ*y*, Δ*h*) were directly measured in the laboratory using a caliper with 0.05-mm accuracy (Table 3). 

Boresight misalignment angles are difficult to directly measure in the laboratory with suitable accuracy, and thus they are usually estimated in an indirect process. With this aim, a bundle adjustment was previously performed using the Structure from Motion (SfM)-based software Agisoft PhotoScan to estimate the camera attitude indirectly, and the platform attitude was directly measured using MPU6050. Because the camera and platform orientation angles are known, the 3 × 3 rotation matrixes between both systems (*R_cam_* and *R_imu_*, respectively) can be computed. Then, the boresight misalignment angles (Δ*r*, Δ*p*, Δ*y*) were computed as presented in Equations (1) and (2), in which *R_b_* is the rotation matrix with boresight angles. Table 3 presents the angular and positional offsets estimated between the Ricoh Theta S (camera 1) and PMTS platform (CAM1/Platform) and between camera 1 and camera 2 (ROP) [16].

(1) $$R_{b}=R_{cam\;}\cdot R_{IMU}^{-1}$$

(2) $$\Delta p=\;\mathrm{asin}[R_{b\;3,1}];\;\Delta r=\;\mathrm{asin}\left[\frac{-R_{b\;3,2}}{\cos\Delta \phi }\right];\;\Delta y=a\cos\left[\frac{R_{b\;1,1}}{\cos\Delta \phi }\right].$$

## 3. PMTS Assessments

### 3.1. Test Areas

**Figure 3:**
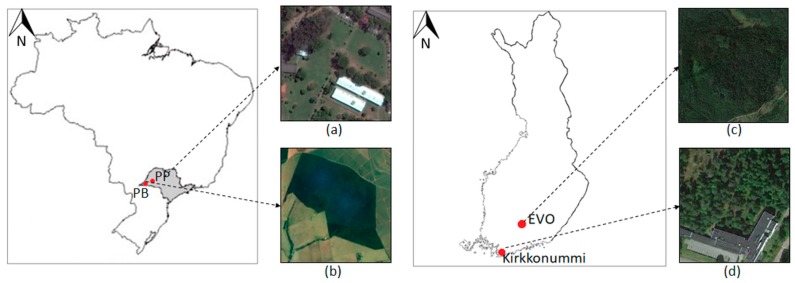
Test areas: (**a**) Test area I—UNESP campus at Presidente Prudente, São Paulo, Brazil; (**b**) Test area II—Ponte Branca at Euclides da Cunha, São Paulo, Brazil; (**c**) Test area IV—boreal forest at Evo, Finland; and (**d**) Test area III—FGI at Kirkkonummi, Finland.

Four different test areas located in Brazil and Finland were used to assess the positional and attitude trajectory (Section 3.2) and the object reconstruction performance using the PMTS data (Section 3.3). Figure 3 illustrates these areas. 

Test areas I and II are located in Brazil. These areas were used to assess the positional accuracy and performance of the Ublox NEO-6M GPS receiver by permitting experiments with distinct canopy coverture density. Test area I (22°07′ S, 51°24′ W) is located inside the São Paulo University (UNESP) campus at Presidente Prudente (PP; Figure 3a). This test site is covered with sparse vegetation areas with *Eucalyptus* trees and ground vegetation (grass). Test area II is an Atlantic tropical forest reserve named *Ponte Branca* (PB), which is located in the West region of São Paulo State (22°25′ S, 52°30′ W; Figure 3b). The phytophysiognomy of the Atlantic Forest biome in test area II is a seasonal semi-deciduous forest, typical of southeast Brazil. This type of vegetation is a transitional forest between coastal forest and non-forest formations comprising trees with a high canopy (e.g., *Ceiba speciosa, Cedrela odorata* and *Handroanthus ochraceus*) and many small trees (e.g., *Syagrus romanzoffiana* and *Plinia cauliflora*). 

Test areas III and IV are located in Finland. These areas were used to assess the attitude measurement accuracy and the object reconstruction performance, respectively. Test area III is located in Kirkkonummi (60°04′ N, 24°32′ E) in the backyard of the Finnish Geospatial Research Institute (FGI) office building (Figure 3d). The area has sparsely distributed mature trees with a tree density of less than 300 stems/ha. Test area IV (Figure 3c) is part of a deciduous boreal forest region at Evo in southern Finland (61°19′ N, 25°11′ E), with a tree density of 625 stems/ha. The forest composition in both areas includes three tree species: Scots pine (*Pinus sylvestris*), Norway spruce (*Picea abies*), and birch (*Betula* sp.).

### 3.2. Assessment of the Positional and Attitude Trajectory Accuracies

#### 3.2.1. Positional Accuracy Experiments

Ublox NEO-6M experiments were performed considering the concepts of absolute and relative positional accuracy [28]. Absolute positional accuracy can be assessed considering the closeness of the measured value to a reference value [28]. To acquire reference positioning data, a Topcon HIPER SR (L1/L2) GNSS receiver was installed in the backpack platform to acquire simultaneously the same trajectory data as Ublox. Topcon SR data were acquired in kinematic mode, and relative positioning in a post-processed mode was computed with RTKlib [21]. The reference data were obtained with 87% of the points with the ambiguity-fixed solution. The standard deviation of the positioning solution was 8.8 mm in N, 9.2 mm in E, and 21.3 mm in height. Ublox NEO-6M data were acquired in NMEA format. 

With the aim of comparative analysis, Ublox NEO-6M and Topcon HIPER SR observations can be synchronized by the GPS time, setting the same collection rate (1 Hz), and determining the physical offset between them in the platform. Therefore, observations were measured by Ublox NEO-6M and Topcon HIPER SR receivers at the same seconds (GPS time) of survey associated to the trajectory. The distance between the coordinates obtained by Ublox NEO-6M and Topcon HIPER SR for the same second of survey, with the subtraction of the offset, can be considered as errors in the trajectory positioning with Ublox NEO-6M. The offsets between receivers were measured directly using a caliper with 0.05-mm accuracy. Planimetric (*D_plan_*) and planialtimetric (*D_planialt_*) errors were analyzed by comparing the distance (D) between point coordinates (East, North and height—E, N, h respectively) measured by the Ublox NEO-6M (*P_ublox_*) and the reference—Topcon HIPER SR (*P_ref_*)—corrected from offsets at a frequency of 1 Hz (Equations (3)–(5)).

(3) $$D=P_{ublox}-P_{ref}+offsets$$

(4) $${\begin{array}{c} D \end{array}}_{plan}=\sqrt{{\left(E_{ublox}-E_{ref}\right)}^{2}+{\left(N_{ublox}-N_{ref}\right)}^{2}}$$

(5) $$D_{planialt}=\;\sqrt{{\left(E_{ublox}-E_{ref}\right)}^{2}+{\left(N_{ublox}-N_{ref}\right)}^{2}+{\left(h_{ublox}-h_{ref}\right)}^{2}}$$

The relative positional accuracy is the closeness of the relative positions of the features in a data set to their respective positions accepted as a reference [28] and provides different insights on the positional accuracy compared to absolute assessments. For the relative positional accuracy assessment, a set of distances between consecutive points acquired with 1 Hz frequency was computed for Ublox NEO-6M (*R_D_*_1(*i*)_) and for Topcon HIPER SR (*R_D_*_2(*i*)_). Then, the differences between the relative distances (Δ*R_D_*_(*i*)_) estimated for both sensors in the same period were compared (Equation (6)). Experiments were performed considering trajectories in open and vegetated areas inside test areas I and II, with the aim of assessing both the positional accuracy and loss of signal in covered areas under simulated forest conditions.

(6) $$\Delta R_{D\left(i\right)}=R_{D1\left(i\right)}-\;R_{D2\left(i\right)}$$

#### 3.2.2. Attitude Accuracy Experiments

Data acquisition for the attitude accuracy experiment was performed in test area III. The absolute accuracy of the PMTS attitude measurement with MPU6050 was evaluated by comparing MPU6050 data to the reference data acquired simultaneously with a more accurate device (VN-100 Rugged IMU), which was also installed in the backpack platform (D*y*; D*p*; D*r*). The estimated boresights misalignment angles between IMUs (Δ*y* = −36°; Δ*p* = 0.26°; Δ*r* = −3.47°) were accounted for in the analysis (Equation (7)). The VN-100 Rugged is a high-performance IMU and Attitude Heading Reference System (AHRS) comprising three-axis accelerometers, three-axis gyros, three-axis magnetometers and a barometric sensor [29]. This IMU has a nominal angular resolution and alignment error of 0.05° degree, which provides good reference data.

(7) $$\;D\gamma =\gamma_{MPU6050}-\gamma_{ref\;}+\;\Delta y;\;D\rho =\rho_{MPU6050}-\rho_{ref}+\Delta p;\;Dr=r_{MPU6050}-r_{ref}+\Delta r\;$$

### 3.3. Assessment of Object Reconstruction

Backpacked measurement systems are very advantageous for forest mapping and enable fast and systematic surveying. These systems can provide detailed and accurate measurements of trees and their surrounding areas from a ground perspective, enabling forest structure analysis that cannot be achieved with any other manual technique or non-terrestrial sensors. This represents a rethinking of the forest survey. In this regard, an example of 3D object reconstruction in a forest environment with the SfM-based software Agisoft PhotoScan [30] using PMTS data was performed. PhotoScan projects were built setting the mathematical model (fisheye), existing IOP (Section 2.1.1), initial EOP from the off-the-shelf navigation system (Section 2.1.2) and system offsets (Section 2.2—Table 3). 

#### 3.3.1. Data Set and Processing 

The PMTS data set, which consisted of dual fisheye video frames and initial EOP, was acquired in test area IV. A set of 66 dual fisheye video frames from test area IV was selected to reconstruct a path in a 16 m × 16 m plot with PMTS. The dual fisheye frames were converted to 132 omnidirectional images (Section 2.1.1), with Ground Sample Distance (GSD) ranging from 3.5 to 7 cm in the central part of the image. Omnidirectional images have a non-uniform spatial resolution, which explains the considerable variation in GSD along the image.

The PMTS data were integrated as described in Section 2.2. Each omnidirectional image has a camera position and attitude collected by the navigation system. The camera position was obtained in 3D geographic coordinates with Ublox NEO-6M. These coordinates were converted to the ETRS-TM35FIN system (E, N, h), the same coordinate system that is used for Ground Control Points (GCP). For those positions in which a loss of signal occurred, camera coordinates and attitude were estimated by an interpolation process. The accuracy values obtained in the assessment of Ublox NEO-6M and MPU6050 were used as standard deviations for the initial EOP data in Photoscan (Section 4.1). 

After project configuration, the PhotoScan workflow consists of: (1) GCP measurement; (2) tie point generation and EOP estimation with a SfM algorithm; and (3) dense image matching for point cloud generation.

1. Six targets to be used as the GCP were installed in some tree stems inside the plot and accurately positioned using a tachymeter (total station). The GCP were targeted with white spheres (Figure 1d). Only small numbers of GCP were available because of the challenges in installing GCP inside the forest due to occlusions and accessibility. The spheres were measured manually in the images.
2. Tie points were generated using the PhotoScan accuracy option “high”, which uses the full resolution images in the processing [30]. Gradual automatic filtering was performed to exclude outliers; the projection accuracy and reconstruction uncertainty methods were used. The errors in the image observations were less than 1 pixel. Finally, a bundle block adjustment was performed to refine the camera positions, camera attitudes and camera calibration parameters, resulting in a final set of oriented images. The final RMSE of the GCPs was 6 cm, which is within the GSD range. Table 4 presents the statistics, including the mean standard deviation and RMSE for the GCPs.
3. A dense point cloud was generated using the multi-view stereo-reconstruction method and the oriented image set. An ultra-high accuracy mode was used that produced, on average, a density with 1.7 cm between points [30,31]. An aggressive depth filtering mode was selected to reduce outliers, particularly those due to the movement of tree leaves and low vegetation.

#### 3.3.2. PMTS Point Cloud Accuracy Assessment 

Different approaches can be used to evaluate point cloud accuracy considering another point cloud as a reference [32]. Comparisons between forest point clouds are challenging because the correspondences and similarity cannot be guaranteed since the environment mapped is not static and the number of outliers can be high. Therefore, one option is to extract a subset of well-defined and stable features in the point clouds, such as trees stems, and then compute the Euclidian distance among them [32]. In other words, the absolute positional accuracy of the PMTS point cloud was evaluated considering how close the features of this cloud were to those of a point cloud used as a reference [28]. The reference data were measured using the Leica ScanStation P40 (Terrestrial LASER Scanner; TLS) in a static survey in the same forest area and reference system and processed in Leica Cyclone 9.0 software, with a 3D positional accuracy of 3 mm at a 50-m range. This was considered an adequate reference since centimetric accuracy is expected for the PMTS point cloud.

The PMTS point cloud was compared to the TLS point cloud using CloudCompare software [33]. Both point clouds were segmented to ensure that only the tree stems were compared. A cross section (clipping box) was defined to extract a delimited section from the point clouds. Automatic filtering using CloudCompare was performed in the delimited section to exclude isolated points, leaves along the stems and remaining outliers. Then, the Euclidian distances between the stems from the PMTS and TLS point clouds were estimated using the least squares local modeling strategy available in CloudCompare [33].

## 4. Results 

### 4.1. Positional and Attitude Trajectory Accuracies

**Figure 4:**
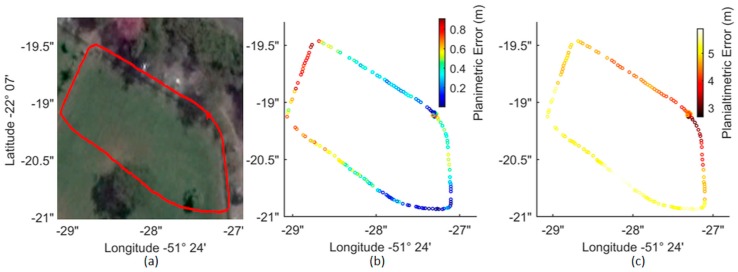
Comparative analyses between the Ublox NEO-6M single-frequency GPS receiver and the dual-frequency GNSS receiver Topcon SR in the open-area trajectory (test area I): (**a**) the open-area trajectory obtained by Ublox (red); and (**b**) planimetric error (D_plan_) and (**c**) planialtimetric error (D_planialt_) in the Ublox trajectory with a one-second interval.

Figure 4 shows the discrepancies between the Ublox NEO-6M and Topcon HIPER SR trajectories (distances between points in both trajectories collected with 1 Hz frequency) obtained in the open-area experiment (test area I—Figure 4a). 

The planimetric discrepancies (D_plan_) varied from 7 to 1 m (Figure 4b). Ublox NEO-6M presented good results for the horizontal coordinates, with an average discrepancy of 34 cm with respect to the Topcon SR positions, a standard deviation of 21 cm and an RMSE of 40 cm. The major problem observed in the Ublox NEO-6M positioning was related to height estimation. When vertical differences were incorporated into the calculation of the 3D point errors (Equation (5)), an average discrepancy of 4.33 m with respect to the Topcon SR positional coordinates was obtained with a 1-m standard deviation and RMSE of 4.45 m. The planialtimetric discrepancies (D_planialt_) are illustrated in Figure 4c; most of the errors were related to the weak altimetric geometry.

Height estimation is a critical issue in GPS positioning because the positioning accuracy significantly decreases due to loss of satellites, multipath errors and poor satellite geometry. For instance, Figure 4c illustrates an error increase in the trajectory estimation near to the vegetated area (left part of the figure), causing signal obstruction and consequently weak observation geometry. It is reasonable to say that such vegetation degraded significantly the GNSS signals tracked from that direction with more impact in the altitude determination. Since height estimation is more affected by the GNSS geometry degradation than the horizontal coordinates, an absolute accuracy varying between 1 and 5 m is expected in height measurement using the Ublox NEO-6M receiver. A potential means of minimizing this problem is to use the height differences between observations instead of the absolute values. The relative positions between observations can be used as constraints in a bundle adjustment [34]. The relative accuracy results showed that for displacements corresponding to 1 s (1 Hz), the differences in the distances (ΔR_D_—Equation (6)) had an average value of 2.6 cm, with a standard deviation of 7.8 cm and a RMSE of 8.2 cm. These results are summarized in Table 5. 

**Figure 5:**
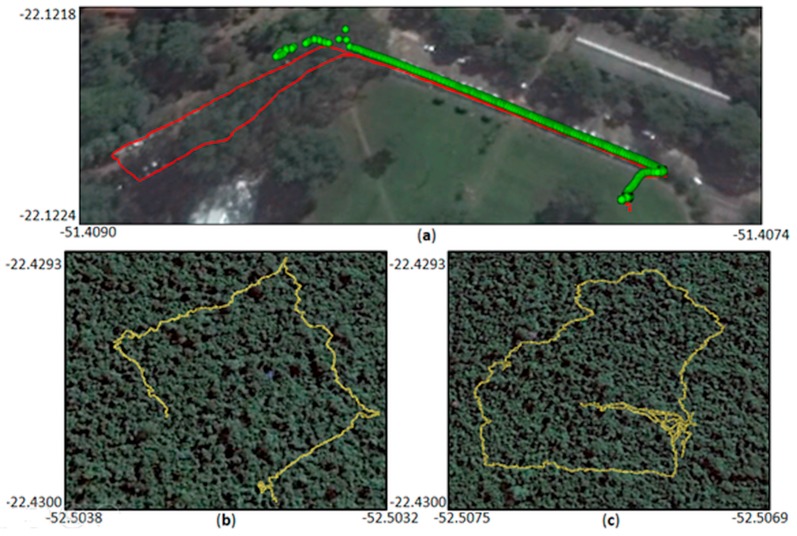
Ublox NEO-6M positioning accuracy: (**a**) Personal Mobile Terrestrial System trajectory in test area I with Ublox NEO-6M (red) and Topcon HiPer SR (green); and (**b**,**c**) examples of the trajectory with Ublox NEO-6M (yellow) inside test area II.

Considering the experiments in the vegetation part of test area I (Figure 5a), major problems were observed in places under forest canopies due to the number of available satellites [35]. Figure 5a shows the trajectory obtained with the Ublox NEO-6M receiver (in red) and the points collected by the Topcon SR receiver (in green). A set of seven data collections was performed in the same area on different days and times. A huge loss of signal occurred in all acquisitions with the Topcon SR receiver in covered areas in which less than the minimum of four satellites were tracked. By contrast, with the Ublox NEO-6M receiver, it was also possible to reproduce the trajectory inside the vegetated area.

This result can be explained by the Topcon SR filter components, which are more rigorous in rejecting low-power signals and non-RHCP (Right Hand Circular Polarization) signals than the Ublox NEO-6M, which acquires more signals, even if of low quality. In addition, the relative positioning, as applied for Topcon SR data, requires simultaneous observations from the base and rover, which is more likely to be performed with less observations than the point positioning used by the Ublox. Therefore, despite the inferior quality, the low-cost receiver linked to the point positioning technique can provide good initial information for reproducing the PMTS trajectory in covered areas. 

Figure 5b,c presents examples of data collection with the Ublox NEO-6M receiver in test area II and show that Ublox NEO-6M reproduced the trajectory with meter-level accuracy even below the high-density canopy. 

**Figure 6:**
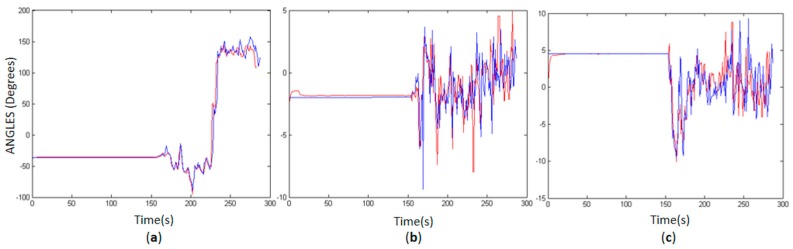
MPU 6050 attitude accuracy, comparing VN-100 Rugged IMU (blue) and MPU6050 (red): yaw (**a**); pitch (**b**); and roll (**c**).

The results of the attitude trajectory assessment are presented in Figure 6, in which the yaw (Figure 6a), pitch (Figure 6b) and roll (Figure 6c) angles measured with the VN-100 Rugged are illustrated by the blue lines (reference) and MPU6050 by the red lines. Despite some outliers, the platform attitude was well reproduced by the MPU6050 data. Since MPU6050 is noise-sensitive, most of the outliers were due to sudden variations in yaw and loss of GPS signal, when the yaw error drift was not corrected. Since the GNSS receiver data has some gaps in covered areas, such as test area III, the accumulated errors in position and attitude calculated from the IMU data increase without a complementary navigation sensor to correct the trajectory drift. 

Table 5 presents the statistics of mean ($\bar{x}$), standard deviation (σ) and RMSE of the discrepancies between attitude angles estimated with VN-100 Rugged and MPU6050 in the same instants of time (1 Hz). In the first approach, outliers were considered in the statistics, which revealed the influence of noise in the attitude estimation (Figure 5). The accuracy decreases mainly in the yaw angle, in which discrepancies between MPU6050 and reference can reach 7°, whereas the pitch and roll errors range between 1° and 3°. However, these results can be improved by combining the disturbance rejection filter with the EKF estimation. In this regard, a simple noise reduction was performed using a moving-average filter, considering as a threshold the RMSE obtained for each angle in the approach without the noise filter. The MPU6050 results are better when outliers are not considered. The RMSE of the discrepancies between MPU6050 and the reference data are approximately 1° for pitch and roll and 3° for yaw.

The Ublox NEO-6M coordinates and MPU6050 data can be used as input data for many photogrammetric processes, such as constraints in the traditional bundle block adjustment. The approach considering navigation data is well known in photogrammetry as the Integrated Sensor Orientation (ISO). For practical purposes, an ISO approach can be used to improve image matching by reducing the search space and the number of wrong matches, reducing the number of GCP, reducing the drift in parameter convergence, and mitigating uncorrected systematic errors [36,37]. 

The accuracy values presented in this Section were used as standard deviations for the input data (EOP) in the object reconstruction process with Photoscan. Therefore, the camera positions were imported in PhotoScan with standard deviations of 4 m and attitude angles with standard deviations of 3°. It is important to point out that it is not clear how the initial position and attitude data obtained from the PMTS are applied in the PhotoScan bundle adjustment. 

### 4.2. Accuracy of Object Reconstruction

**Figure 7:**
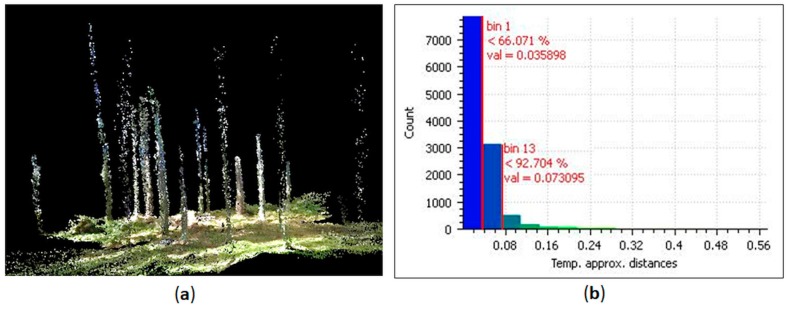
Accuracy assessment of object reconstruction: (**a**) Personal Mobile Terrestrial System point cloud after tree leaf filtering; and (**b**) histogram of the estimated errors in tree positions.

Figure 7 illustrates the object reconstruction results, which show (Figure 7a) the filtered point cloud generated with PMTS data and (Figure 7b) the histogram of the PMTS point cloud classified in four classes according to the Euclidian distance from the TLS reference. 

The accuracy of object reconstruction of the tree stems (11,910 points) was assessed by comparing the PMTS cross section to the TLS reference. The Euclidian distances between points of the tree stems in the PMTS point cloud and in the reference were calculated (Figure 7b). From this set, 66% of the points had errors less than 3.5 cm (7861 points), consistent with the average GSD of the images; 15.6% of the errors (1853) were within the range of 3.5 to 5 cm, and 10.8% of the errors were within the range of 5 to 7 cm (1293). Therefore, the errors in the stem points acquired by the PMTS were close to the image GSD range in 92.4% of the points, when considering the normal distribution; 7.6% of the points (904) had errors larger than 7 cm. The statistics of the average and standard deviation of the discrepancies between the PMTS and reference were 3.4 cm and 3.7 cm, respectively, comparable to the values for other PMTS approaches for forest mapping [7,8,9].

## 5. Discussion

The aim of the experimental assessments was to analyze the feasibility of the developed PMTS for forest mapping. This section presents a discussion of PMTS performance compared to other backpacked approaches for forest applications that use optical systems [9,10] or LASER scanners [7,8,11]. 

In optical systems, the level of accuracy that can be achieved is directly related to the GSD and intersection geometry, which depends on the sensor size, focal length, sensor-object distance and image scale. Omnidirectional images have a reduced angular resolution and a larger image scale variation than perspective images; however, the increased field of view allows more features to be matched between images, thereby reducing the number of images required to map the environment and enabling a fast and systematic survey. For instance, the optical approach with five perspective cameras in a side-by-side configuration proposed by Forsman et al. [9] is limited by system weight (13 kg) and the implementation of a single-camera method considering differences in perspective between cameras. Only a limited area is acquired from the center of the plot. Therefore, a PMTS with omnidirectional system represents an alternative to improve the limitations pointed out in [9] and achieve compatible accuracy. With this motivation, Tseng et al. [10] presented an omnidirectional system composed of eight single-lens cameras in back-to-back position. This system provided a panoramic image resulting from a stitching process. Therefore, techniques for perspective images, such as the traditional bundle adjustment, can be applied. However, the stitching process can include errors, such as blurring, and further computational cost. Our PMTS preserves the original geometry of the Ricoh Theta S images to avoid additional errors in the photogrammetric process.

The main advantages of the Ricoh Theta S in relation to the systems developed by Forsman et al. [9] and Tseng et al. [10] are its low weight and cost. However, this compact solution has a drawback: low image resolution. The results showed that due to this low image resolution, the reconstruction accuracy is higher for trees near the trajectory. The imaging range of the system with acceptable accuracy is 5 m. Objects farther than 5 m from the imaging system were not reconstructed or were inaccurate. This is the main limitation of the PMTS compared to LASER approaches [7,8,11]. This issue can be solved by changing the Ricoh Theta S to another omnidirectional camera with higher resolution. The methodology presented in the paper can be extended to any other omnidirectional camera model.

Advantages with respect to LASER systems can also be mentioned. The use of initial information (IOP, EOP and ROP) combined with a minimum number of GCPs permits an accurate trajectory estimation (centimetric level), even in covered areas. This is the main advantage of optical systems compared to LASER PMTS approaches. Most LASER approaches have been assessed in boreal forests [7,8], which have sparse tree distributions. Therefore, dense forests, such as test area II, create new challenges for PMTS application, especially in terms of trajectory estimation. Indirect methods for the determination of EOP, such as image bundle adjustment, are a good alternative for forest mapping, especially in dense areas. Direct methods based on GNSS and IMU may not be compatible with the required accuracy, especially in cases of signal loss [23]. Furthermore, a direct determination requires robust and high-accuracy equipment, which, in general, means high costs. However, there are also critical challenges in the use of passive optical systems in forest environments. Forest, especially the dense one, has a huge illumination variation and shadow occurrence in the images. The presence of shadows can negatively affect image quality and photogrammetric processes, including image matching, image classification, automatic detection of objects and loss of information, which requires an image pre-processing step [38]. Therefore, it is also important to highlight the possibility of combining an omnidirectional system with independent georeferencing capability with other complementary technologies, such as a LASER scanner. For instance, an optical system can complement the LASER scanner in position and attitude trajectory estimation, planimetric accuracy and assigning RGB colors for LASER point clouds. In addition, the LASER scanner can increase the mapping range area, penetrate vegetation with good accuracy in depth, and is subject to fewer problems in shadow areas and homogeneous areas, such as indoor environments.

With respect to PMTS accuracy, most of the PMTS mentioned in Section 1 obtained a centimetric-level of accuracy in 3D object reconstruction ranging between 2 and 5 cm. The object reconstruction results showed that the PMTS point cloud has an average accuracy of 3.5 cm. Therefore, the system presented in this paper can be considered consistent with other PMTS approaches [7,8,9,10], especially when compared to other optical systems [9]. 

## 6. Conclusions 

A low-cost and lightweight PMTS comprising an omnidirectional camera with off-the-shelf navigation systems was presented and assessed in this study. Experiments explored the feasibility of the proposed PMTS for forest mapping, a significant application of backpacked systems.

Our study was the first to evaluate a low-cost GPS receiver and MEMS-IMU in a backpack system in a forested environment. Our results provide new insights on the suitability of these novel sensors, which have recently entered the market. Despite positioning challenges in forest areas, for instance, due to multipath, signal obstruction and measurement noise, the GPS point positioning using UBLOX NEO-6M reproduced the PMTS trajectory with centimetric accuracy in planimetry and meter-level accuracy in altimetry. These data can be used, for instance, as a source of initial information in a traditional bundle adjustment. Another interesting possibility for the application of low-cost GPS data is to estimate the relative displacements between observations along the trajectory, which can be used as relative constraints in the bundle adjustment. However, the use of MEMS-IMU is more challenging, mainly because the measurement accuracy decreases quickly as a function of time due to deterministic and random errors. Particularly in forests, where GPS data are not constantly available, these errors increase without a trajectory drift correction. More rigorous methods for modeling these errors and filtering noises are required.

Second, our study was the first to assess the performance of a low-cost backpack-mounted omnidirectional camera system in the estimation of tree stem parameters. Centimetric accuracy was obtained for object reconstruction using the PMTS data processed with PhotoScan software. These results are comparable to those for other PMTS approaches for forest mapping, which are currently ten to hundreds of times more expensive. Modeling of individual tree stems is a fundamental process for extracting DBH measurements and other forest parameters along the stem, which would be extremely laborious to measure manually, thus highlighting a potential application of this system. 

Based on our results, we can identify some drawbacks related to the PMTS that provide scope for future research. (1) The 8-s time gap between successive still images with the Ricoh Theta S prevents the dynamic sequential acquisition of images, leading to the decision to use frame videos with low resolution in our system. Therefore, this technique can be extended for other dual systems, such as the Samsung Gear 360, LG 360 and Nikon KeyMission 360. (2) Omnidirectional images do not follow a perspective geometry, which requires modifications of classic photogrammetric models. (3) Due to the image resolution, the imaging range of the system is less than 5 m, and thus more strips are required to cover the area than for LASER scanner PMTS approaches. (4) Further investigations should be performed to evaluate the effects on PMTS navigation system data of using an integrated sensor orientation approach, with rigorous analysis of the stochastic properties of the data. (5) Further experiments considering larger test areas are also suggested. 

## Figures and Tables

**Table 1:** Ricoh Theta S digital camera and fisheye lens settings.

Settings	Camera Ricoh Theta S
Sensor Size	Two 1/2.3” CMOS Sensors (14 Mpx)
Still Image	2688 × 2688 pixels in each sensor
Dual Fisheye Video	960 × 1080 pixels in each sensor
Principal Distance	1.43 mm
Camera Dimensions	44 mm × 130 mm × 22.9 mm—weight 125 g
Fittings	Remote control, RSWC201 wireless, HDMI and USB.

**Table 2:** Main technical specifications of MPU 6050 and Ublox NEO-6M.

**MPU6050**	**Sensor**	**Full Scale Range**	**Resolution**	**Linearity**	**Sensitivity**
	Accelerometers	±4 g	0.001198 m/s^2^	0.5%	8192 LSB */g
	Gyroscopes	±250°/s	0.0076294°/s	0.2%	131 LSB */(°/s)
**UBLOX NEO 6M**	**Sensor**	**Time pulse frequency range**	**Time pulse resolution**	**Velocity resolution**	**Heading resolution**
	GPS L1 frequency	0.25 Hz to 1 kHz	30 ns	0.1 m/s	0.5°

* LSB (Least Significant Bit per unit).

**Table 3:** Angular and positional offsets in the Personal Mobile Terrestrial System.

Offsets	Δ*x* (mm)	Δ*y* (mm)	Δ*h* (mm)	Δ*r* (°)	Δ*p* (°)	Δ*y* (°)
CAM1/Platform	101.4	85.05	138.0	−78.75	29.45	174.57
ROP	−0.79	0.41	1.904	0.0149	0.0526	179.66

**Table 4:** Mean ($\bar{x}$), Standard deviation (*σ*) and RMSE of GCP estimation after bundle adjustment.

Statistics	E (m)	N (m)	H (m)	Total Error (m)
$\bar{x}$	0.007	0.0006	−0.001	0.007
*σ*	0.059	0.021	0.004	0.063
RMSE	0.060	0.021	0.005	0.064

**Table 5:** Summary of the results for the navigation sensors: (1) Mean ($\bar{x}$), Standard deviation (σ) and RMSE of discrepancies between MPU6050 and the reference data with a noise filter for yaw, pitch and roll angles and (2) $\bar{x}$, σ and RMSE of discrepancies between UBLOX NEO 6M and the reference data in the absolute and relative positional accuracy assessments.

**MPU6050**	**Euler Angles**	$\bar{x}$ **(*°*)**	**σ (*°*)**	**RMSE (*°*)**
	Yaw (γ)	−0.0888	3.0652	3.0588
	Pitch (ϕ)	0.0190	0.5474	0.5463
	Roll (ω)	0.0999	1.0690	1.0710
**UBLOX NEO 6M**	**Accuracy**	$\bar{x}$ **(m)**	**σ (m)**	**RMSE (m)**
	Absolute (Planimetric)	0.34	0.21	0.40
	Absolute (Planialtimetric)	4.34	1.0	4.45
	Relative (Planialtimetric)	0.026	0.078	0.082
